# Effect of Modified Polyvinyl Alcohol Fibers on the Mechanical Behavior of Engineered Cementitious Composites

**DOI:** 10.3390/ma12010037

**Published:** 2018-12-22

**Authors:** Mian Sun, Youzhi Chen, Jiaoqun Zhu, Tao Sun, Zhonghe Shui, Gang Ling, Haoxuan Zhong, Yourui Zheng

**Affiliations:** 1 State Key Laboratory of Silicate Materials for Architectures, Wuhan University of Technology, Wuhan 430070, China; 18202762912@sina.cn (M.S.); cyzly@whut.edu.cn (Y.C.); zhujiaoq@whut.edu.cn (J.Z.); zhshui@whut.edu.cn (Z.S.); linggang0609@163.com (G.L.); 18011134689@163.com (H.Z.); 15150565301@163.com (Y.Z.); 2 School of Materials Science and Engineering, Wuhan University of Technology, Wuhan 430070, China

**Keywords:** engineered cementitious composites (ECC), polyvinyl alcohol, fiber modification, mechanical behavior

## Abstract

Polyvinyl alcohol (PVA) fiber was proposed to enhance the mechanical performance of engineered cementitious composite in this research. A mixture of engineered cementitious composite with better expected performance was made by adding 2% PVA fiber. Mechanics tests, including pressure resistance, fracture resistance, and ultimate tensile strength, were conducted. They reveal that the engineered cementitious composites not only exhibit good pressure resistance, but they also exhibit excellent fracture resistance and strain capability against tensile stress through mechanics tests, including pressure resistance, fracture resistance, and ultimate tensile resistance. To further improve the engineered composites’ ductility, attempts to modify the performance of the PVA fiber surface have been made by using a vinyl acetate (VAE) emulsion, a butadiene–styrene emulsion, and boric anhydride. Results indicated that the VAE emulsion achieved the best performance improvement. Its use in fiber pre-processing enables the formation of a layer of film with weak acidity, which restrains the hydration of adjacent gel materials, and reduces the strength of transitional areas of the fiber/composite interface, which restricts fiber slippage and pulls out as a result of its growth in age, and reduces hydration levels. Research illustrates that the performance-improvement processing that is studied not only improves the strain of the engineered cementitious composites, but can also reduce the attenuation of the strain against tensile stress.

## 1. Introduction

Due to cementitious composites’ advantages of convenient construction, fine performance, and cost-saving, they are the most used building materials since their introduction in the 19th century [1]. Nevertheless, the innate disadvantages of inorganic non-metallic materials, including high fragility, low strain, and cracking, have led to multiple issues, and have limited their further development [2]. Specifically, it is the lack of ductility of cementitious composites, and their resultant ultimate loading, that causes fragile cracks and damage due to their lack of durability under normal loading and their lack of sustainability, which have constrained the applications of cementitious composites [3]. Hence, to ensure the harmonious co-existence of artificial facilities and the natural environment, and the reduction of damage to humans due to the collapse of buildings, it is increasingly urgent that concrete possessing high ductility, high extensibility, high durability, and high sustainability is developed for the construction of buildings [4].

At present, the fibers added are all millimeter-grade steel fibers. The steel fiber has a clear reinforcing effect on concrete and can effectively reduce the occurrence of large cracks, but it is not ideal to use the steel fiber to reduce the occurrence of small cracks. Small-scale fibers such as polyhexene fibers and polyvinyl alcohol fibers can restrain the expansion of micro-cracks from some micro-crack sources and prevent the generation of some micro-cracks, so that the crack width becomes smaller, which stops them from being connected [5]. Polymer fibers with a lower modulus of elasticity can reduce the stress concentration of concrete generated due to original defects, which plays a toughening role [6].

Yu K. et al. [7] selected polyethylene fibers and then mixed them into concrete to enhance the tensile load capacity of concrete substrates. High polymer and high-strength polyethylene fibers provide an enough fracture bridging capacity, which significantly improves the tensile toughness of the concrete matrix.

Ezio Cadoni et al. [8] conducted a dynamic tensile test on PVA-FRC (polyvinyl alcohol fiber reinforced concrete) to test fiber-reinforced concrete notched specimens at three different strain rates (50 s, 100 s, and 200 s) using a modified Hopkinson rod device. The experimental result shows that, as the strain rate increases, the tensile strength is significantly improved, and the fracture energy and ultimate deformation are significantly reduced.

M. Haskett et al. [9] studied the compression zone and tension zone of PVA fiber-reinforced concrete and found that PVA fiber in the compression zone effectively inhibited the cracking of concrete and slowed down the damage of the internal structure. Acing as a bridge, the fiber transmits the tensile stress in the tension zone, so PVA fiber reinforced concrete exhibits excellent ductility and a good performance after cracking.

Blanco et al. [10] conducted a comparative experiment to study the effects of different fiber types on the performance of concrete slabs and carried out finite element simulations, which found that different fiber types (steel fibers, plastic fibers) using general design methods led to large differences in the simulation experiment. Therefore, they put forward correction factors for different fiber types in the FRC board design. Therefore, the modification of the interface among PVA and steel fiber and the concrete matrix also requires different modification methods.

Engineered cementitious composites are a type of high-performance fiber-reinforcing composite concrete material that are systematically designed and optimally configured by employing theories in micromechanics, fracture mechanics, and statistics [11]. Through the configuration of the performance and relationship between chopped fibers, cementitious composites, and the transitional area of the interface, dramatic strain and strain-hardening characteristics are derived from the gain of a relatively low volume fraction of fibers [12]. Fabricated engineered cementitious composites should possess the distinctive characteristics of multiple cracks and strain hardening. They should be able to generate only tiny cracks and continuous load bearing when receiving tensile stress and bending stress, and eventually reach above a 3% ultimate strain against tensile stress [13]. The composition of engineered cementitious composites includes fibers, an emulsion, ultrafine sand (generally less than 0.3 mm), water, a highly effective water reducer, and a density-increasing stabilizer. The ratio between water and emulsion is generally less than 0.45 and voluminous auxiliary gel materials such as fly ash are used and the fiber-volume fraction is not greater than 2.5% [14]. Li, V.C. et al. [3] proved that they have radical strain and higher energy-consumption capabilities compared to normal fiber-enforced cementitious composites. Therefore, they will be significantly utilized in improving the ductility of the structure, the energy consumption, and the shock resistance. Cementitious composites have broad developmental potential in the applications of huge transformational structures, anti-shock structures, renovation structures, anti-seismic structures, and road paving structures.

Theoretical research on engineered cementitious composites commenced in 1999 [15]. This is the earliest proposition for adding polyethylene fibers into cementitious composites of high strength and high molecular weight, to achieve the reinforced strength and ductility of cementitious composites. Li and Kanda applied polyvinyl alcohol into cementitious composites, and made PVA-ECC (PVA fiber reinforced engineered cementitious composite) [16]. Young’s modulus and the tensile resistance strength level of PVA fibers are higher than those of general cementitious composites. PVA fibers are able to increase the strength of concrete, and it does not form clusters in the process of stirring. Currently, both domestic and international studies on engineered cementitious composites focus on PVA fibers [17].

The first crack strength of engineered cementitious composites is equal to that of general cementitious composites when receiving tensile stress, but the ultimate tensile strain is approximately 500–1000 times that of the latter, which exhibit the characteristics of strain and ductility at a high magnitude [18]. The significant strain capability against the tensile stress of engineered cementitious composites at the tensile stress–strain curve is extremely similar to that of metallic materials during the process starting from cracking and peak loading to the nullity of the structure as a whole, presenting a pattern of strain hardening. Engineered cementitious composites have changed in fragility compared to traditional cementitious composites and other cementitious composites, and they have overcome an array of disadvantages caused by fragility due to the unique characteristics of multiple cracks, strain-toughness, and superbly powerful ductility [19].

Kanda et al. [20] found that, when the shear span ratio is 1, under the conditions of the non-provision of reinforcing bars, the engineered cementitious composites’ shearing resistance loading is exponentially higher than that of the concrete beam. The corresponding distortion capability increases by 225% and exhibits a failure mode with eminent extensibility. Fischer Gregor et al. [21] conducted periodical circulated shearing resistance tests for reinforced concrete and reinforcing bars-engineered cementitious composite compound beam elements. Results suggest that the shearing failure mode of reinforcing bars/engineered cementitious composites is similar to that of the failure mode of extensibility. Numerous tiny slant cracks occur, and anti-shearing ductility remarkably outperforms that of reinforced concrete. In addition, Siad H. [22] found that engineered cementitious composites exhibit moderately high self-healing abilities. Self-healing abilities improve the engineered cementitious composites’ working life in a changing environment.

Engineered cementitious composites exhibit good environmental protection performance. More than 600 million tons of fly ash—particles left after the burning of coal—are yielded globally each year [23]. Preventing the ash from flowing into the air, and disposing of the dust particles is a troublesome issue. In the last century, academics have found that the ash can be a partial replacement for cement, to reduce the volume of gel materials in building materials, and to consume the dust particles. In general, normal cementitious composites use 10%–25% fly ash to substitute for cement, whereas engineered cementitious composites use approximately 40%–60% fly ash, whereby superior environmental protection performance is achieved [24].

Normal cementitious composites very often burst under high temperatures [25]. This is mainly due to fire causing the structure’s temperature to rise rapidly, which densifies the hydrated products of the cementitious composites in concrete, the structure of which prevents the vapor from escaping. Therefore, substantial vapor pressure is stored in the internal micro-holes of the concrete. When the pressure exceeds the weaker ultimate tensile resistance strength of the cementitious composites, a sudden crash is caused. Organic PVA or PE (polyethylene) fibers in engineered cementitious composites will melt in the high temperature of fire and form paths for vapor and the release of the vapor pressure in elements of engineered cementitious composites, and prevents the cementitious composites as a whole from cracking. As a result, they possess good fire resistance and high temperature resistance capabilities [26].

The paper applies the design principles of engineered cementitious composites as part of the preliminary aims of studying additive agents’ impacts on the functional performance of engineered cementitious composites, and optimizes the composition to gain a slurry with good fluidity. Based on this, it aims to make engineered cementitious composites with good performance. This paper discusses the impacts of the processing methods of the fiber surface on the transitional area of the interface, and the ductility of cementitious composites, by which an optimized processing method is sought, to further improve its ductility, on the basis of making engineered cementitious composites. The impact of processing methods for the fiber surface on the transitional area of the interface, and the ductility of cementitious composites is studied.

## 2. Materials and Methods

### 2.1. Raw Materials

The cement used for this research is PO (ordinary portland cement) 42.5 cement, made by Huaxin Cement Co., Ltd. (Huangshi, China), with a relative density of 3141 kg/m^3^ (refer to Table 1 and Table 2 for chemical analysis and technical specifications). The fly ash used was first-grade ash made by China Railway Major Bridge Engineering Group Co., Ltd. Ninth Company (Nanjing, China) where d(0.5) is 11.6, d(0.9) is 43.5 (refer to Table 1 for detailed chemical elements, d is diameter). The fine aggregate used was quartz sand, with a fineness of 100 mesh, which means that the diameter per particle was 0.15 mm. A highly effective water reducer called polycarboxylate superplasticizer (made by Wuhan Geruilin Building Materials Technology Co., Ltd., Wuhan, China) was used. The mother liquor is white transparent thick liquid, and the solid content is 40%, which is diluted to 20% for practical use.

The polyvinyl alcohol fibers (PVA fibers) used for the study encapsulated two types, and respectively, they are high-strength high elastic modulus PVA fibers (fabricated by Sinopec Chongqing SVW (Sichuan Vinylon Works) Chemical Co., Ltd., Chongqing, China), and REC 15 model PVA fibers (fabricated by Kuraray Co., Ltd., Tokyo, Japan). They were chopped to 12 mm on average (refer to Table 3 for concrete performance specifications).

The vinyl acetate emulsion (hereinafter named ‘VAE emulsion’) that was used for the study is the new model VAE CW40-905 (fabricated by Sinopec Chongqing SVW Chemical Co., Ltd., refer to Table 4 for detailed performance specifications).

The hydroxyl propyl methyl cellulose (HPMC) used for the study is HK-type, including 50,000 and 150,000 viscosity (refer to Table 5 for technical specifications). Note that, due to the viscosity of the HPMC being high, and its quantity of use being low, the dried powder does not easily scatter evenly during the process of stirring. Therefore, the HPMC was made into a 1% solution in advance, and the impact of water was omitted in its volume of water content.

The boric anhydride used for the study is made by Sinopharm Chemical Reagent Co., Ltd. (Beijing, China). Therein, the purities are as follows: sulfate (SO_4_) ≤0.02%, heavy metal (calculated by Pb) ≤0.005%, silicon and alkali metal content (calculated by SO_4_) ≤0.1%, and purity ≥98.0%. The butylbenzene emulsion was supplied by the Sinopharm Chemical Reagent Co., Ltd. (Beijing, China), model no. JNYS-BS-8825GH.

### 2.2. Specimen Preparation

#### 2.2.1. Ratio of the Mixture

The fundamental proportions of the engineered cementitious composites included cement, fly ash, and quartz sand. Per 500 g, the water–binder ratio that was adopted was either 0.3 or 0.35, and a 2% (2% fiber mixing amount can ensure ECC has a tensile strain of 3% to 7%. When the fiber mixing amount exceeds 2%, although the tensile properties of the material increase with the increase of the mixing amount, it cannot guarantee sufficient workability. In addition, it may result in fiber agglomeration and uneven dispersion [27].) The volume fraction of PVA fibers was added as well as a small quantity of HPMC density-increasing stabilizer and a highly effective polycarboxylate superplasticizer. Standard maintenance of the sample was adopted. The HPMC and the fraction of the water reducer were adjusted, accordingly, as in the results and discussion, so that cementitious composites with good fluidity could be obtained.

#### 2.2.2. Pre-Processing of the Fibers

The method of pre-processing the fibers was as follows:

- VAE emulsion: Water was added to 50 g VAE emulsion to form a 400 mL volume of diluted solution in a 1 L beaker. The solution was stirred evenly, and an ultra-sonicator (TOSO25-52, Nanjing Toso Ultrasonic Cleaning Machine Co., Ltd, Nanjing, China) was applied to scatter the particles. Pre-calculated and pre-weighed PVA fibers was added and soaked under constant stirring, with the simultaneous application of the ultra-sonicator for 10 min. Afterwards, the fibers were removed and placed on a mesh to be baked at low temperature until it was half-dry. Water was used to wash away the extra emulsion and scattered particles, and the fibers were re-baked for use.
- Cement and boric anhydride: A total of 5 g boric anhydride was made up to a 300 g solution in water, stirred evenly with 100 g cement, and formed into a very thin cement slurry. A pre-calculated and pre-weighed amount of PVA fiber was added and evenly mixed. Then it was removed and placed on a mesh to be baked at a low temperature for use.
- Butyl-benzene emulsion and boric anhydride: A total of 5 g boric anhydride was made up to a 350 g solution with water, and 50 g butyl-benzene emulsion was added with mixing. The subsequent processing method was similar to that of Step a. for processing the VAE emulsion. The derived pre-processed fibers were then ready for use.

#### 2.2.3. Mixing Process 

The method of the mixing process was as follows:

- The preparation of the mortar without fibers. (According to the ASTM C305–14 [28]).
- Add the fibers or modification fibers.
- Repeat Step A and cast it in two layers.
- Strip the mold after 24 to 48 hours and then put them in the standard curing condition.

### 2.3. Experimental Methods

#### 2.3.1. Experiment on Rheological Properties

The experiments on rheological properties used the slurry without the fibers added. The study adopted a rotor-type viscosity meter to test the rheological properties of the cementitious composite slurry. An R/S-SST model of a soft-solid rheological property testing instrument (manufactured by Brookfield Co., Ltd., New York, NY, USA) was used, and it was paired with a V 80-40 propeller rotor. The scope of the shearing stress tested was 6–200 Pa, and the shearing velocity could be of an infinitely variable speed within 0–1000 rpm. The time of the shearing velocity was set as:

a) Under 200 s^−1^ constant shearing velocity, with the test conducted on changes in the apparent viscosity at 0 min, 30 min, and 120 min after the slurry was made. The time for each test was 60 s.
b) Within 180 s, the rotor’s shearing velocity was increased constantly from 0 s^−1^ to 200 s^−1^, and the test changes of the apparent viscosity, along with the shearing velocity of different slurries at 1 min, 31 min, and 121 min, and the yield viscosity of the slurry, were calculated according to the Rheo V2.8 program (version 2.8, Brookfield Co., Ltd., New York, NY, USA).
c) Within 180 s, the rotor’s shearing velocity was increased constantly from 0 s^−1^ to 200 s^−1^. Afterwards, in the same period of time, the rotor’s shearing velocity was evenly decreased from 200 s^−1^ to 0 s^−1^.

The relationship of the shearing stress, along with the shearing velocities of different slurries at 1 min, 31 min, and 121 min, and the thixotropies of different groups of slurries, were judged as per the area outlined by the curve [29].

#### 2.3.2. Test on the Strength of Pressure Resistance and Fracture Resistance

According to the Testing Methods of Strength of Cement Mortar (ISO Methods) [30], an electrical hydraulic universal testing machine (model: WDW 10, supplied by Wuxi Jianyi experiment instrument Co., Ltd., Wuxi, China) was applied to make a sample of mortar and test the strengths of pressure resistance and fracture resistance.

#### 2.3.3. Test on Tensile Performance

**Figure 1:**
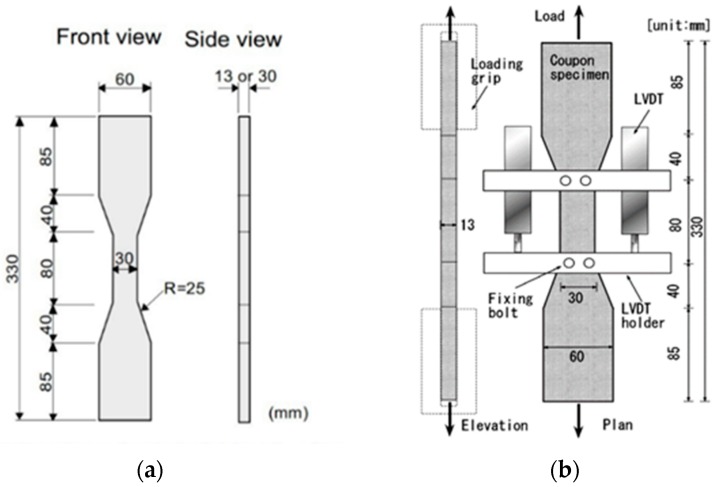
The specimen and device of the tensile test. (**a**) Tensile specimen; (**b**) Diagram of the tensile loading device

**Figure 2:**
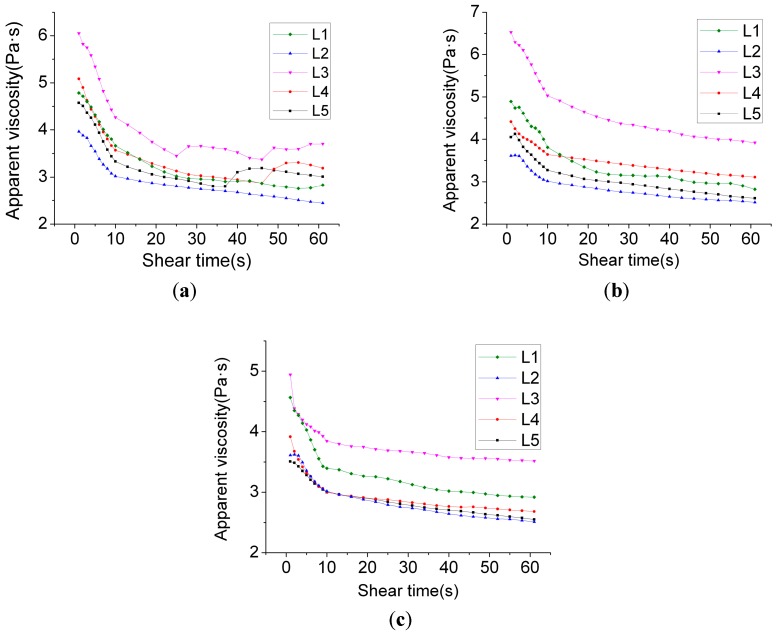
Relationship between the slurry shear rate and the apparent viscosity at a constant shear rate. (**a**) Constant shearing velocity test (hydration for 0 min); (**b**) Constant shearing velocity test (hydration for 30 min); (**c**) Constant shearing velocity test (hydration for 120 min).

The equipment used for the tensile performance test was an Instron 5882 electrical universal material testing machine (Instron, Boston, MA, USA). The test was implemented in an environment of 25 °C at 75% humidity using self-made tensile clamps (Figure 1) as per the design and construction recommendations for HPFRCC materials, set out by JSCE (Japan Society of Civil Engineers) [31]. The tensile loading device is shown in Figure 2, with a pair of clamps at the top and the bottom. After the sample for tensile testing was ready, aluminum LVDT (linear variable differential transformer) clamps were configured and fixed securely at the tensile area of the sample. The test loaded the tensile test sample through the shift control method, and adopted a 0.4 mm/min loading velocity. Shift sensors (model: SCAH series, Abek Sensors Co., Ltd., Beijing, China) were located at the two sides of the sample test data of tensile stress, and transmitted information to the Keithley 2700 data collection apparatus (Keithley 2700, Keithley Instruments, Beaverton, OR, USA). The data for tensile stress and stress were collected per second. When processing the data, the average value was used for the data, as derived from the shift sensors at two channels.

#### 2.3.4. Test on Micro-Performance 

A field emission scanning electron microscope (Quanta 450 FEG, manufactured by FEI, Hillsboro, OR, USA) was used for the observation and analysis of the scanning electron microscopy (SEM) shape and its appearance in the study. Resolution capability: ≤3 nm@1 kV, largest pixel: 6144 × 4096, range of magnification: 10–10,000,000 times. SEM was used for viewing the shape and appearance of the hydrated products of the cementitious composites, the shape and appearance of the transitional area at the interface of the cementitious composites and the fibers, and the shape and appearance of sections after the fibers were pulled [32].

## 3. Results and Discussion

### 3.1. Study on the Rheological Properties of the Slurry

The slurry has outstanding rheology and stability properties, and it ensures that the engineered cementitious composites gain good functional performance and stability, including fluidity, anti-segregation, thixotropy, and thixotropy loss, and more [33,34,35].

Based on the extensibility test in Table 6, L2, L4, and L5 exhibited good fluid extensibilities. The detailed rheological properties of each group are illustrated and discussed, according to the subsequent rheological instruments. 

The apparent viscosity of slurry is influenced by the scope of the shearing stress effects, and the temperature and composition of the materials, along with the trend of change of the shearing stress. The apparent viscosity reflects various points of information, including the yield stress and the degree of the hydration of cement [36,37]. Figure 2 exhibits the slurry’s laws of change for the apparent viscosity–shearing time curve, along with the time, at different hydrating times. It can be observed from Figure 2a that compared with L1, where only 0.9% water reducer was added, the apparent viscosity of group L2 by mixing 1.8% water reducer was relatively lower, and it was located underneath the L1 curve.

By mixing 10% HPMC of 50,000 viscosity in group L3, the viscosity of the slurry further increased. This was due to the varying molecular weights of HPMC of different viscosities, and also the differing lengths of the molecular chains. For the addition of long-chain molecular HPMC, its branches’ composite clusters will absorb water molecules, which further influence the superplasticizer and cement particles. The overlap and intersection of HPMC molecules change the types of clustering of particles on a small scale in the former slurry, and causes the viscosity of the slurry to dramatically increase. In the meantime, it can be observed that an HPMC of 150,000 viscosity produced greater density-increasing effects than HPMC of 50,000 viscosity, but the effects were not significant. From Figure 2, it was found that the relative distribution position of the curve was similar to that of Figure 2b. In the process of the constant shearing velocity test, the types, quantities, and volumes of the water reducer for HPMC generated an impact on the apparent viscosity–shearing velocity relationship, and the effects weakened in sequence. When the hydrating time reached 120 min, L2, L4, and L5’s viscosity curves primarily overlapped. Nevertheless, at a later stage, a difference among the three emerged. The regularities of distribution corresponded to the quantities and types of HPMC. The viscosity of L2 using 5% HPMC at 50,000 viscosity was the lowest while the viscosities of L4 using 10% HPMC at 50,000 viscosity, and of L5 using 5% HPMC of 150,000 viscosity, were relatively higher.

**Figure 3:**
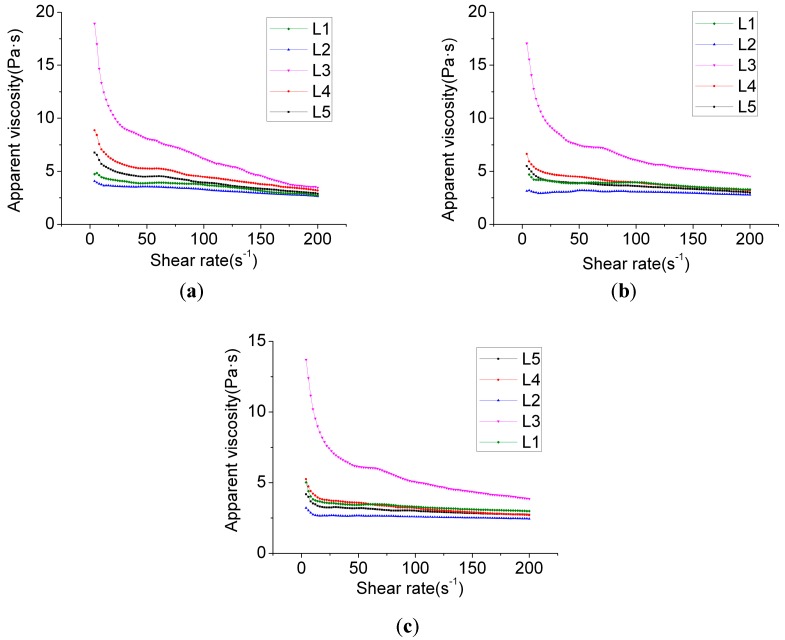
Apparent viscosity changes over time and changes with the shear rate. (**a**) Accelerated shear test (hydration for 1 min); (**b**) Accelerated shear test (hydration for 31 min); (**c**) Accelerated shear test (hydration for 121 min).

Figure 3 shows that group L3’s apparent viscosity was very low when the shearing velocity was moderately low. However, when the shearing velocity was relatively high, the apparent viscosity rapidly decreased. This suggests that stability of the slurry was poor. Other groups exhibited good stabilities, and the trend of the shearing viscosity decreasing along with the increase of the shearing velocity was moderately mild. This was associated with the equilibrium of effects between the HPMC and the water reducer. Once the long-chain molecule HPMC is added, on the one hand, the composite clusters on the chain that are in proximity to water molecules absorb the water molecules and prevent them from floating upwards. On the other hand, through the absorption of cement particles and the flocculent hydrated product, the clustered cement particles sink and fall, and the proportion of hydrated gel is reduced. Collisions between the particles are reduced. The slurry’s stability thus significantly improves. Nonetheless, when excessive HPMC slurry is added, due to the large quantity of long chain-shaped substances, the viscosity of the slurry is excessively great. However, when the shearing frequency is moderately high, due to the HPMC, long chains disaggregate into irregular coil structures. The viscosity of the slurry thereby rapidly falls. Similarly, L4’s change of viscosity was the outcome of long-chain disaggregation. Nevertheless, under the conditions of increase in the volume of the water reducer, the viscosity at a low shearing velocity was low. By analyzing L1 and L2’s laws of change, it was observed that HPMC significantly influences the viscosity at an early stage while, along with the HPMC’s disaggregation at a later stage, the difference of viscosity that is brought about by different types of HPMC slowly decreases. In contrast, different quantities of HPMC influence the viscosity at high levels of shearing stress. Such a difference is closely related to the volume of water reducer. In other words, the effects of shearing stress on the disaggregation of HPMC are rather limited, and when the quantity of use significantly increases while the volume of water reducer remains unchanged, the viscosity at different shearing stress levels increases.

**Figure 4:**
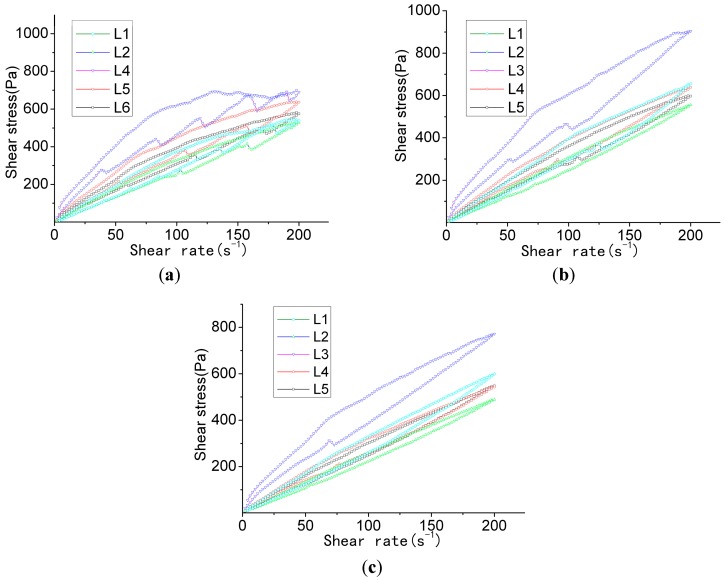
Testing of thixotropy. (**a**) Thixotropy test (hydration for 1 min); (**b**) Thixotropy test (hydration for 31 min); (**c**) Thixotropy test (hydration for 121 min).

By comparing Figure 2 and Figure 3, and by analyzing the apparent viscosity–shearing time curve at different hydration times in the same group, it can be found that the apparent viscosity reduces along with the extension of hydration time, rather than increasing along with the extension of hydration time. This is possibly due to the chosen polycarboxylate superplasticizer taking effect upon the slow release, and its water-reducing effect gradually becoming distinctive over a period of time after its addition. Therefore, the outcome of apparent viscosity reduction, along with the extension of the hydration time, occurs. Based on the thixotropy test analysis chart and test results (Figure 4), it can be revealed that the thixotropy index generally decreases along with the extension of hydration time, which indicates that the stability of the slurry gradually increases. Moreover, at the stage of the gradual fall of shearing velocity, the fluctuation of the curve gradually decreases. This is congruent with the thixotropy test results. To some extent, thixotropy is able to reflect the changes in the internal structure of the slurry. The greater the thixotropy, the more severe the damage to the flocculation structure that is formed in the slurry.

Test results shown in Table 7 also revealed that the thixotropy index of group L2 was moderately low at all points in time. It was, respectively, 9910.953 Pa·s^−1^ at 1 min, 7673.119 Pa·s^−1^ at 31 min, and 5063.676 Pa·s^−1^ at 121 min, with the change of thixotropy being small. After 30 min, the thixotropy index dropped to 2237.834 Pa·s^−1^. After 120 min, the thixotropy index dropped to 4847.277 Pa·s^−1^. Regularities of change were also evident. Therefore, in follow-up experiments, group L2’s composition was applied as the benchmark to be implemented in subsequent experiments.

### 3.2. Mechanics Performance

The strength level of the engineered cementitious composites made for the study was 40 MPa (refer to Table 8 for parameters). As such, group C0 was the base group without mixtures of the fibers. Group C1 mixed a 2% volume fraction of REC-15 PVA fibers. Group C2 mixed 2% PVA fibers pre-processed by VAE emulsion. Group C3 mixed boric acid and PVA fibers processed by cement.

The water–binder ratio for each group of cementitious composites was 0.35, with a 28 d strength-of-pressure resistance near 40 MPa, a strength-of-fracture resistance near 15 MPa, and a strength-of-fracture resistance for groups mixed with fibers, which was higher by more than 1 over the C0 base group without mixing in fibers. Nevertheless, the strength-of-pressure resistance of the C0 base group without mixing in fibers was the highest among all of the groups. The strength-of-pressure resistance of group C1 mixing 2% PVA fibers only achieved 40.6 MPa with a decrease of approximately 4 MPa, whereas its strength-of-fracture resistance achieved 18.2 MPa, which was far greater than group C0’s 5.7 MPa. The changes of the two strengths may have been caused by the mixture with the fibers. On the one hand, the PVA of high strength and a high elasticity modulus was able to robustly increase the moderately low tensile resistance and bending resistance of the cementitious composites. On the other hand, a 2% volume fraction of PVA fibers would be significantly influential on the dense structures of cementitious composites since numerous tiny air holes would be brought in during the process of stirring, and these are difficult to remove in the process of vibration. Thus, this led to the evident increase of the strength-of-fracture resistance and the decrease of the strength-of-pressure resistance over a small scale.

Compared to group C1 without pre-processing, the strength-of-pressure resistance and the fracture resistance of group C2 using PVA fibers pre-processed by VAE emulsion increased over a small scale. This may be due to a layer of VAE emulsion film covering the surfaces of the PVA fibers. Through the weak-acid organic film, the hydration of the cementitious composites at the surface area of the fibers would be affected. As a consequence, the fiber strength and adhesive strength would drop slightly, which would mitigate the possibility of the fibers being pulled to be broken by cementitious composites during earlier loading. As a result, the fibers’ slippage friction and reinforcing effects are fully utilized.

The strength of group C3, which used PVA fibers processed by boric anhydride and cement slurry, exhibited an evident decrease. Compared with group C1, which used unprocessed PVA fibers, its strength of pressure resistance was only 34.2 MPa, and the strength-of-fracture resistance was only 12.5 MPa. The introduction of boric anhydride creates a moderately significant impact on the strength of the engineered cementitious composites. In contrast, if viewed from a percentage strength attenuation, its impact on the strength-of-pressure resistance was rather small, and its impact on the strength-of-fracture resistance was greater. When the fibers are processed by boric anhydride and cement, the adhesion between the particles on its surface and on the fibers is not firm, and some of the boric anhydride and cement particles will fall off in the process of stirring, and mix with the cementitious composites. Boric anhydride has serious effects on the hydration of cement. Thus, the overall strength-of-pressure resistance of the cementitious composites dropped by 25.7%. Moreover, due to the boric anhydride content being the greatest on the surfaces of the fibers, the effect on the cementitious composites near the interface is the most significant. Excessively low strength and insufficient friction between the fibers and the composites lowers the ductility of the system. Hence, the attenuation of the strength-of-fracture resistance of group C3 reached only 12.5 MPa.

When boric anhydride and a cement slurry are used to process the fibers, due to the adhesion of the slurry, and due to the fibers not being firm, particles will fall off in the process of stirring, and this will cause the overall alkalinity of the cementitious composites to be insufficient and affect hydration. This leads to a decrease in strength and results in poor effects. The acidity of the VAE emulsion is rather weak, and it has a moderately low impact on the alkalinity of the fiber/composite interface. Due to the VAE emulsion not mixing with the boric anhydride, this will cause the VAE emulsion to solidify. Therefore, a follow-up attempt has been made by mixing a butadiene–styrene emulsion into boric anhydride to process the fibers. In addition, an attempt has been made by adjusting the water–binder ratio of the cementitious composites to improve their strength. Refer to Table 9 for important influential factors in the composition and for the test results of strength.

**Figure 5:**
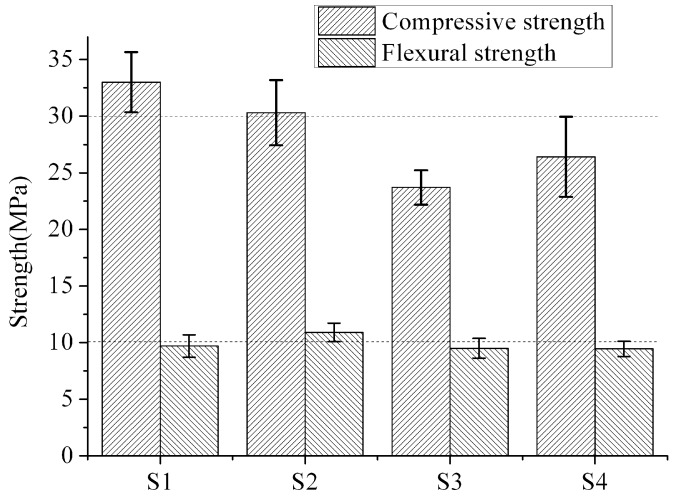
Compressive and flexural strength of 7d ECC.

It was found from the test results (Figure 5) of the sample with 7 d maintenance, that the strength-of-pressure resistance of group S1 with 0.3 water–binder ratio was higher than that of any of the other groups with 0.35 water–binder ratio, which reached 33.0 MPa. Nonetheless, differences in the strength-of-fracture resistance at an early stage in the groups were insignificant. For groups with 0.35 water–binder ratio, the strength-of-pressure resistance and the fracture resistance of group S2 using VAE emulsion processing was higher than the two groups without fiber processing or that used boric anhydride and butadiene–styrene emulsion processing. This indicates that the fibers with VAE emulsion processing take effect upon reinforcement. The strength-of-pressure resistance of group S3, which used a butadiene–styrene emulsion and boric anhydride processing, created a certain degree of attenuation, and the impact on the strength-of-fracture resistance was small. This may be caused by the release of boric anhydride onto the surfaces of the fibers.

**Figure 6:**
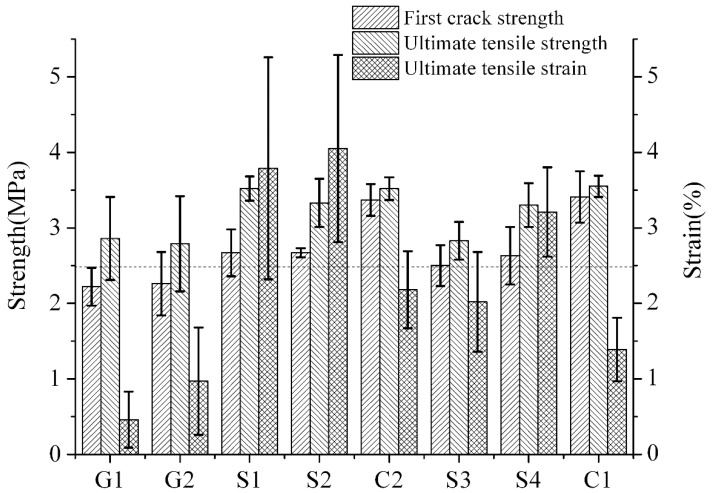
Tensile strength and strain of ECC.

Table 10 and Figure 6 show the compositions of the tensile samples, and the important influential factors of each group. The test results of the samples in groups G1, G2, and group S were differences in 7 d tensile strength and strain rate.

Groups G1 and G2 used domestic PVA fibers (manufactured by Sinopec Chongqing SVW Chemical Co., Ltd., Chongqing, China), and the water–binder ratio was 0.3. The PVA in group G2 was soaked in boric anhydride–butadiene–styrene emulsion diluent for 10 min prior to use, and it was removed and baked at low temperature until it was half-dry. Afterwards, extra emulsion was washed away by water, and the PVA was re-baked for use. A polymer film with acidity will form after processing of the surfaces of the fibers. Due to the domestic fibers’ surfaces not being processed, –C–OH composite clusters on the surfaces of the PVA fibers can form firmly combined hydrogen bridges with –OH in the cementitious composites [38]. In addition, the diameter and the strength-of-tensile resistance of a single thread of domestic fiber are weaker than that of the imported fibers. All of these factors increase the probability of the occurrence of fractures at the early stage of cracking in the cementitious composites, and increase their inability to transmit stress and or to utilize better strain capabilities. Regarding the pre-processing of fibers with acid polymer films, on the one hand, the films on the surfaces of the fibers effectively reduce the firm adhesion of the composite clusters that are in proximity to water and the cementitious composites. On the other hand, the acidity of the film will reduce the strength of the transitional area at the fiber–cement interface. Thus, the fibers are able to achieve better slippage-pulling results. It makes more significant use of the fibers’ deformation and further improves the ductility of the engineered cementitious composites.

**Figure 7:**
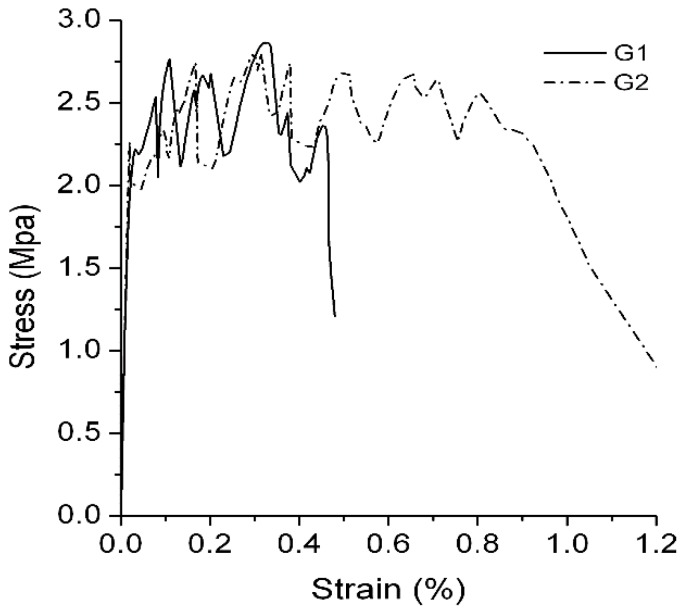
Stress-strain curve of G1 and G2.

It is revealed by comparing the test results (Figure 7) of group G1 and group G2 that a layer of acid polymer film that is attached to the surfaces of the fibers comes into significant effect for the ultimate tensile strain of the engineered cementitious composites: with the same compositional proportions and with different types of fibers, the tensile strain increased from 0.46% in group G1 to 0.97%, which is an increase of approximately 110.8%.

**Figure 8:**
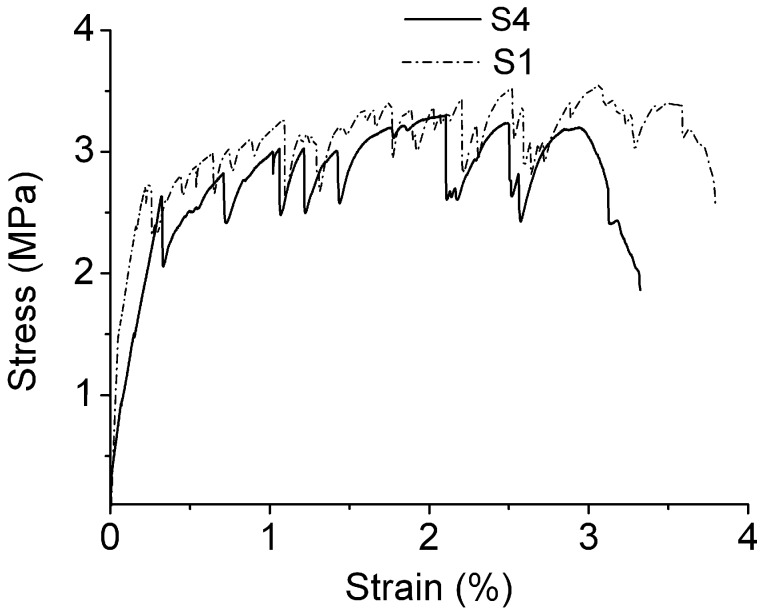
Stress-strain curve of S1 and S4.

Group S1’s water–binder ratio was 0.3, and it was mixed with a 2% volume fraction of PVA fibers imported from Japan. After maintenance for 7 d, the strength of the first crack was 2.67 MPa. The ultimate tensile strength was 3.52 MPa and the strain achieved was 3.79%. Group S4’s water–binder ratio was 0.35. After maintenance for 7d, the strength of the first crack was 2.63 MPa, the ultimate tensile strength was 3.3 MPa, and the ultimate tensile strain was 3.21%. Both of the two groups exhibited certain strain-hardening abilities (Figure 8). This indicates that group S1, with a lower water–binder ratio, exhibits a moderately higher tensile strength and a greater strain capability. Nevertheless, due to a great gap in fluidity, this study chose a composite proportion of 0.35 water–binder ratio with superior functional performance. As a matter of fact, in accordance with V. C. Li’s research findings, it is not necessarily true that the lower the water–binder ratio for engineered cementitious composites is, the better. Under the premise that the strength level requirement is satisfied, a greater water–binder ratio can be adopted to achieve a higher strain capability.

**Figure 9:**
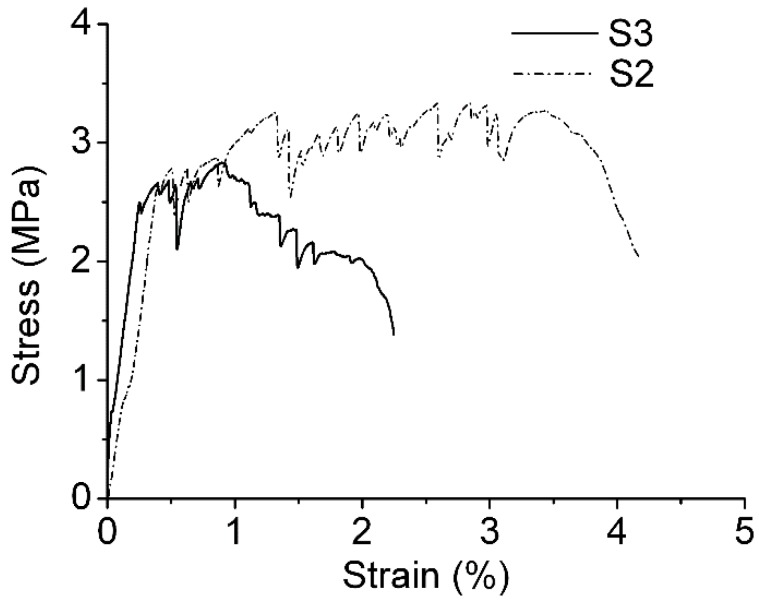
Stress-strain curve of S2 and S3.

Figure 9 exhibits group S2, which adopted the VAE emulsion for modification, and its water–binder ratio was 0.35. After maintenance for 7 d, the first crack strength tested was 2.67 MPa, the ultimate tensile strength was 3.33 MPa, and the ultimate tensile strain was 4.05%. Compared with group S4 with the same composition and using unprocessed REC-15 fibers, their strengths were fundamentally the same, but the ultimate tensile strain increased by 26.2%. The test results clearly suggested that, by applying the VAE emulsion, a film with weak acidity formed on the surfaces of the fibers, and the strength of the composites near the interface decreased while the overall strength of cementitious composites was not affected. The fibers are not easily pulled to be broken in the process of stretching, and the ductility of the engineered cementitious composites improved. 

The fibers used in group S3 were processed by a diluent of boric anhydride and butadiene–styrene emulsion. Its first crack strength was 2.5 MPa, the ultimate tensile strength was 2.83 MPa, and the ultimate tensile strain was 2.02% (Figure 9). Compared with other groups, its first crack strength and ultimate tensile strength were slightly lower while its strain capability dropped more significantly. As shown in Table 11, compared with base group S4, group S3 dropped by 37.1%. Compared with group S2 with VAE emulsion processing, it only had half the strain value. Furthermore, based on the stress–strain curve, group S3 exhibited strain softening during testing.

**Figure 10:**
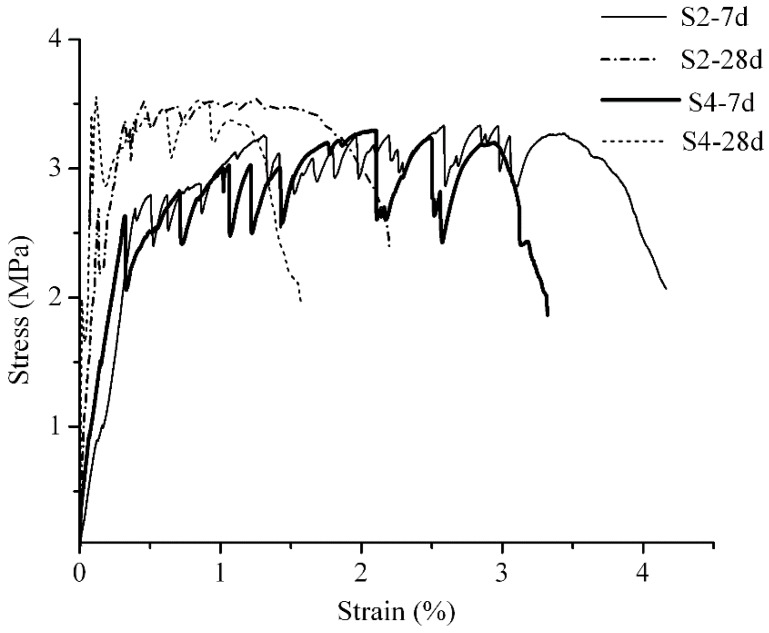
Stress-strain curve of S4 and S2.

Compared with group S4, which used REC-15 PVA fibers, and group S2, which used modified PVA fibers (Figure 10), it can be found that group S3’s first crack strength gained relatively significant increases in 7 d, 28 d strengths (28 d strength is the test result for both group C1 and group C2), which increased from approximately 2.6 MPa at 7 d to approximately 3.4 MPa at 28 d (the first peak value was abnormal, and the second peak value was used in the average). When the first crack occurs in the material, the strain is extremely small, and the stress is borne by cementitious composites. Therefore, the first crack strength can be taken as the tensile resistance strength of the cementitious composites. It mainly reflects the hydration process and the nature of the cementitious composite materials. After the occurrence of the first crack, the fibers at the cracks come into play. They transmit force to the composites that are not cracked, which are characterized by multiple cracks and strain hardening, and derive the peak value of tensile stress. Thus, the ultimate tensile stress of engineered cementitious composites mainly relates to the fibers used, and after maintenance for 28 d, the gains in ultimate tensile strength only increase over a small scale.

The dramatic change in the figure is the ultimate tensile strain. After maintenance for 28 d, the ultimate strain of the general group decreased from 3.21% to 1.39%, and that of the modification group decreased from 4.05% to 2.18%. The attenuations were, respectively, 56.7% and 46.2%. Along with the growth of age and the development of hydration in the cement, not only did the strengths of the composites increase, but the transitional areas of the fiber/composite interfaces became closer. Hence, the tensile slippage of the fibers in the composites is more greatly constrained, and they are more easily broken when pulled. Therefore, the strain capability attenuates radically. Other academics’ findings also suggest that, for engineered cementitious composites, the peak value of the tensile strain capability occurs at an age of approximately 10 d.

This study implemented modifications through the pre-processing of the surfaces of the fibers. A layer of acid film was attached onto the surfaces of the PVA fibers that were in proximity to water. Thereby, the firm adhesion between clusters that are in proximity to the water molecules and the cementitious composites is mitigated. Moreover, the acidity of the film influences the hydration of the gel materials near the fibers/composites, which reduces the strength of the transitional area of the fiber–cement interface, so that the fibers are able to achieve better slippage–pulling out results. The fibers’ deformation is, thus, utilized more significantly and the ductility of the engineered cementitious composites is further enhanced. Hence, the 7 d tensile strain of the modification group was 126.2% that of the general group. The 28 d tensile strain was 156.8% that of the general group. Under the conditions of long age and full hydration, the attenuation rate for the tensile strain of the modification group was 10.5% lower when compared to the general group without modifications on the fibers.

### 3.3. Microstructure

**Figure 11:**
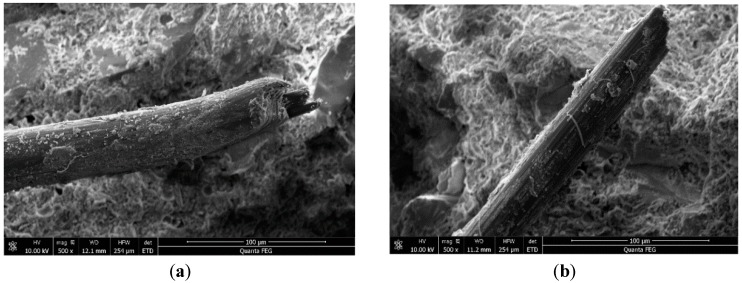
Microstructure of S4 and S2. (**a**) Untreated PVA fiber of S4; (**b**) Pretreated fiber of S2

It was found, based on the micro-appearance from SEM, that some gel materials were attached to the surfaces of the fibers in group S4 (Figure 11a), and there were instances of slight ‘neck-shrinking’ phenomena at the breaking points. This suggests that cracking occurs when the fibers in the cementitious composites slip slightly. Small quantities of gel materials are attached onto the surfaces of the fibers in group S2 (Figure 11b). When there is evident slippage on the surface, the friction with the cementitious composites generates axial damage. This indicates that emulsion processing improves the effects of slippage through which strain capability is enhanced.

**Figure 12:**
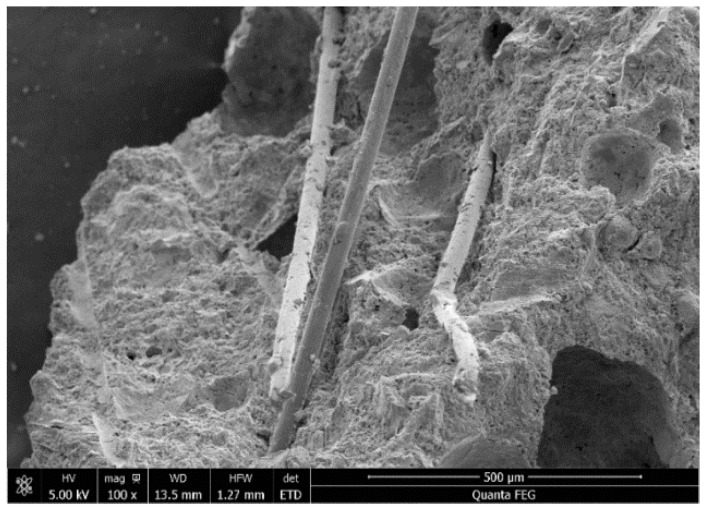
Microstructure of S3.

Group S3 exhibited strain softening during testing. It was found via analysis of its SEM micro-appearance (Figure 12), that the PVA fibers processed by a diluent of boric anhydride emulsion have a weak binding ability with the cementitious composites, and only a small amount of large fragments was found. It was also found from the scanning results that the pulled fibers were moderately long and complete. This explains why pulling the fibers out from the cementitious composites was rather easy since this may have been due to the weak strength of the cementitious composites near the surfaces of the fibers, which was caused by the acidity of the boric anhydride. The above-mentioned impacts leading to the strain capability of the fibers were not utilized, and the strain capability of group S3 was poor.

## 4. Conclusions

This paper investigates the impact of additive agents on the functional performance of engineered cementitious composites, so that a slurry with good fluidity is optimized to form engineered cementitious composites with good performance. The main content of the discussion are concerned with the impact of the processing methods of the fiber surface on the transitional area of the interface and the ductility of the cementitious composites, and the impact of the processing methods of the fiber surface on the transitional area of the interface and the ductility of the cementitious composites. The major results of the research are summarized as follows.

(1) By using HPMC and a polycarboxylate superplasticizer to collectively configure the slurry, a slurry with good performance can be made. By mixing gel materials, including 1.8% water reducer and 0.05% HPMC, the viscosity and the thixotropy of the slurry reach a minimum, and the rheology of the slurry achieves a fine state.

(2) Under the condition that the water reducer is unchanged, using an HPMC of higher viscosity or adding HPMC volume will remarkably increase its initial viscosity. Nevertheless, by accounting for the effects of shearing, its viscosity rapidly decreases and eventually comes to the state of being close.

(3) PVA fibers with modification by a VAE emulsion enable the formation of a film with weak acidity, by which a better pulling–slippage outcome is achieved. Engineered cementitious composites that are made using processed fibers primarily do not affect the strength of the composites. However, an ultimate tensile strain of 4.05% is achieved at 7 d, which is 126.2% that of the PVA fiber groups without processing. This implies that the ductility of the engineered cementitious composites can be further effectively improved.

(4) A film with weak acidity that is added onto the fibers by pre-processing with a VAE emulsion is able to restrain the hydration of the gel materials near the fibers, and reduce the strength of the transitional area at the fiber/composite interface. Furthermore, the restriction of fiber slippage is caused by greater age, and is deepened by the hydrating process. During the time period of 7 d and 28 d, the tensile strain of the engineered cementitious composites with non-modified PVA fibers drops from 3.21% to 1.39%, and that of the modification groups drops from 4.05% to 2.18%, which attenuates by 56.7% and 46.2%. Furthermore, the attenuation rate decreases by 10.5%. This suggests that modification processing not only improves the strain capability, but it also reduces the impact of the tensile strain.

(5) Using a diluent of boric anhydride and butadiene–styrene emulsion also enables the formation of an acid film. Nonetheless, due to boric anhydride having a moderately high impact on the hydration of the gel materials, and its partial release during the process of stirring, the outcomes of the strength of the cementitious composites decreases, the adhesion between the fibers and the cementitious composites is too loose, and the fibers can be easily pulled out. The fibers’ deformation ability cannot be utilized. This causes a decrease in the ultimate tensile stress and strain. However, it also implies that its effects on the configuring interface are eminent. The impact of the volume of boric anhydride on cementitious composites needs further study.

## Figures and Tables

**Table 1:** Chemical analysis of cement and fly ash.

Oxide	SiO_2_	Al_2_O_3_	Fe_2_O_3_	CaO	MgO	K_2_O	Na_2_O	SO_3_	Loss
Cement	21.6	5.85	2.84	59.80	2.24	0.67	0.21	2.06	3.70
Fly ash	46.43	38.02	3.11	7.51	0.23	0.89	0.34	0.68	2.78

**Table 2:** Technical specifications of cement.

Stability	Setting Time (min)		Flexural Strength (MPa)		Compressive Strength (MPa)		Specific Surface Area (m^2^/kg)
	Initial	Final	3d	28d	3d	28d	
Qualified	193	273	4.3	6.7	22.6	43.4	342

**Table 3:** Performance indexes of PVA fiber.

Type	Diameter (μm)	Aspect Ratio	Tensile Strength (MPa)	Tensile Elastic Modulus (GPa)	Ultimate Elongation (%)
Domestic PVA	31	387	1400	35	6.5
REC15	40	300	1560	42	7.0

**Table 4:** Performance indexes of VAE.

Solid Content (%)	Viscosity (mPa·s)	PH	Ethylene Content (%)	Dilution Stability	MFFT (°C)
54.4	1500–2500	4.0–6.5	≤5.0	≤3.5	≤-3

(MFFT: Minimum film forming temperature).

**Table 5:** Performance indexes of HMPC.

Nominal Viscosity (mPa·s × 10^4^)	Methoxyl Content (%)	HydroxyPropyl Content (%)	Water Content (wt%)	Ash Content (wt%)	Density (g/cm^3^)	Fineness (Mesh)
5/15	17.0–24.0	4.0–12.0	≤5.0	≤1.0	1.26–1.31	>80

**Table 6:** The result of the slurry spread.

No.	Water Reducer (%)	HPMC		Slurry Spread (mm)
		Dosage (%)	Viscosity (mPa·s × 10^4^)	
L1	0.9	5	5	295
L2	1.8	5	5	325
L3	0.9	10	5	245
L4	1.8	10	5	310
L5	1.8	5	15	315

**Table 7:** Thixotropy of the slurry.

No.	Testing Time (min)	Thixotropy (Pa·s^−1^)
L1	1	11,260.893
	31	13,936.090
	121	9248.396
L2	1	9910.953
	31	7673.119
	121	5063.676
L3	1	17,469.007
	31	21,300.803
	121	14,922.739
L4	1	15,206.990
	31	11,401.008
	121	7677.211
L5	1	12,476.844
	31	10,988.725
	121	6695.922

**Table 8:** Compressive and flexural strength of 28d mortar.

No.	PVA(%)	Others	Compressive Strength (MPa)	Deviation (%)	Flexural Strength (MPa)	Deviation (%)
C0	0	None	44.4	-	5.7	-
C1	2	None	40.6	100.0	18.2	100.0
C2	2	VAE	42.6	105.0	19.1	105.0
C3	2	Boric anhydride + cement	34.2	84.3	12.5	68.7

**Table 9:** Compressive and flexural strength of 7d mortar.

Number	Water-Binder Ratio	Others
S1	0.30	None
S2	0.35	VAE
S3	0.35	Boric anhydride + butylbenzene emulsion
S4	0.35	None

**Table 10:** Mixture ratio of tensile testing.

No.	Water-Binder Ratio	Others	First Crack Strength (MPa)	Ultimate Tensile Strength (MPa)	Ultimate Tensile Strain (%)
G1	0.35	Domestic PVA	2.22	2.86	0.46
G2	0.35	Domestic PVA + VAE	2.26	2.79	0.97
S1	0.3	-	2.67	3.52	3.79
S2	0.35	VAE	2.67	3.33	4.05
S3	0.35	Boric anhydride + butylbenzene emulsion	2.5	2.83	2.02
S4	0.35	-	2.63	3.3	3.21

**Table 11:** Tensile properties of S4 and S2.

No.	First Crack Strength (MPa)	Ultimate Tensile Strength (MPa)	Ultimate Tensile Strain (%)
S2-7d	2.67	3.33	4.05
S2-28d	3.4	3.4	2.18
S4-7d	2.63	3.3	3.21
S4-28d	3.2	3.3	1.39

## References

[1] Valvona F., Toti J., Gattulli V., Potenza F. (2017). Effective seismic strengthening and monitoring of a masonry vault by using Glass Fiber Reinforced Cementitious Matrix with embedded Fiber Bragg Grating sensors. Compos. Part B-Eng..

[2] Huang B., Li Q., Xu S., Zhou B. (2018). Tensile fatigue behavior of fiber-reinforced cementitious material with high ductility: Experimental study and novel P-S-N model. Constr. Build. Mater..

[3] Li V. (2007). Progress and Application of Engineered Cementitious Composites. J. Chin. Silic. Soc..

[4] Mo K.-H., Loh Z.-P., Tan C.-G., Alengaram U.-J., Yap S.-P. (2018). Behaviour of fibre-reinforced cementitious composite containing high-volume fly ash at elevated temperatures. Sadhana-Acad. Proc. Eng. Sci..

[5] Kasagani H., Rao C.-B.-K. (2018). Effect of graded fibers on stress strain behaviour of Glass Fiber Reinforced Concrete in tension. Constr. Build. Mater..

[6] Irshidat M.-R., Al-Shannaq A. (2018). Using textile reinforced mortar modified with carbon nano tubes to improve flexural performance of RC beams. Compos. Struct..

[7] Yu K., Wang Y., Yu J., Xu S. (2017). A strain-hardening cementitious composites with the tensile capacity up to 8%. Constr. Build. Mater..

[8] Cadoni E., Meda A., Plizzari G.-A. (2009). Tensile behaviour of FRC under high strain-rate. Mater. Struct..

[9] Haskett M., Mohamed Sadakkathulla M., Oehlers D., Guest G., Pritchard T., Sedav V., Stapleton B. Adelaide Research and Scholarship: Deflection of GFRP and PVA fibre reinforced concrete beams. Proceedings of the 6th International Conference on FRP Composites in Civil Engineering (CICE2012).

[10] Blanco A., Pujadas P., De la Fuente A., Cavalaro S.H.P., Aguado A. (2016). Influence of the type of fiber on the structural response and design of FRC slabs. J. Struct. Eng..

[11] Nezerka V., Hrbek V., Prosek Z., Somr M., Tesarek P., Fladr J. (2018). Micromechanical characterization and modeling of cement pastes containing waste marble powder. J. Clean. Prod..

[12] Bicer K., Yalciner H., Balks A.-P., Kumbasaroglu A. (2018). Effect of corrosion on flexural strength of reinforced concrete beams with polypropylene fibers. Constr. Build. Mater..

[13] Yu J., Li H., Leung C.-K.-Y., Lin X., Lam J.-Y.-K., Sham I.-M.-L., Shih K. (2017). Matrix design for waterproof Engineered Cementitious Composites (ECCs). Constr. Build. Mater..

[14] Khan M.-Z.-N., Hao Y., Hao H., Shaikh F.-U.-A., Liu K. (2018). Mechanical properties of ambient cured high-strength plain and hybrid fiber reinforced geopolymer composites from triaxial compressive tests. Constr. Build. Mater..

[15] Harrison P.-T., Levy L.-S., Patrick G., Pigott G.-H., Smith L.-L. (1999). Comparative hazards of chrysotile asbestos and its substitutes: A European perspective. Environ. Health Perspect..

[16] Kanda T., Li V.-C. (2006). Practical Design Criteria for Saturated Pseudo Strain Hardening Behavior in ECC. J. Adv. Concr. Technol..

[17] Huang B., Li Q., Xu S., Liu W., Wang H. (2018). Fatigue deformation behavior and fiber failure mechanism of ultra-high toughness cementitious composites in compression. Mater. Des..

[18] Ding Y., Yu K., Yu J., Xu S. (2018). Structural behaviors of ultra-high performance engineered cementitious composites (UHP-ECC) beams subjected to bending-experimental study. Constr. Build. Mater..

[19] Altwair N.-M., Johari M.-A.-M., Hashim S.-F.-S. (2012). Flexural performance of green engineered cementitious composites containing high volume of palm oil fuel ash. Constr. Build. Mater..

[20] Kanda T., Lin Z., Li V.-C. (2000). Tensile stress-strain modeling of pseudostrain hardening cementitious composites. J. Mater. Civ. Eng..

[21] Fischer G., Li V.-C. (2002). Effect of matrix ductility on deformation behavior of steel-reinforced ECC flexural members under reversed cyclic loading conditions. ACI Struct. J..

[22] Siad H., Lachemi M., Sahmaran M., Mesbah H.-A., Hossain K.-A. (2018). Advanced engineered cementitious composites with combined self-sensing and self-healing functionalities. Constr. Build. Mater..

[23] Hussein A.-A.-E., Shafiq N., Nuruddin M.-F., Ramli M.F., Junoh A.K., Roslan N., Masnan M.J., Kharuddin M.H. (2015). Compressive Strength and Interfacial Transition Zone of Sugar Cane Bagasse Ash Concrete: A Comparison to the Established Pozzolans. AIP Conference Proceedings.

[24] Kapoor K.-M.-E., Singh S.-P., Singh B. (2016). Durability of self-compacting concrete made with Recycled Concrete Aggregates and mineral admixtures. Constr. Build. Mater..

[25] Chousidis N., Ioannou I., Rakanta E., Koutsodontis C., Batis G. (2016). Effect of fly ash chemical composition on the reinforcement corrosion, thermal diffusion and strength of blended cement concretes. Constr. Build. Mater..

[26] Li Q., Gao X., Xu S., Peng Y., Fu Y. (2016). Microstructure and Mechanical Properties of High-Toughness Fiber-Reinforced Cementitious Composites after Exposure to Elevated Temperatures. J. Mater. Civ. Eng..

[27] Li V. (1992). Conditions for Pseudo Strain-Hardening in Fiber Reinforced Brittle Matrix Composites. Appl. Mech. Rev..

[28] (2014). Standard Practice for Mechanical Mixing of Hydraulic Cement Pastes and Mortars of Plastic Consistency.

[29] Lu H., Xie C., Gao Y., Li L., Zhu H. (2015). Cement Slurries with Rheological Properties Unaffected by Temperature. SPE Drill. Complet..

[30] (2009). Cement Test Methods Determination of Strength.

[31] Pellegrino C., D’Antino T. (2013). Experimental behaviour of existing precast prestressed reinforced concrete elements strengthened with cementitious composites. Compos. Part B-Eng..

[32] Shaikh F.-U.-A. (2013). Deflection hardening behaviour of short fibre reinforced fly ash based geopolymer composites. Mater. Des..

[33] Atlasov R.-A., Nikolaeva M.-V., Popov B.-I. (2017). Analysis of results of casings cementing in the presence of absorption bands on the example of Mastakhskoye gas-condensate field and Nedzhelinskaya area. Neftyanoe Khozyaistvo.

[34] Demirci E., Wojtanowicz A.-K. (2016). Pilot size process visualization: Gravity fluid displacement method to stop annular gas migration. J. Nat. Gas Sci. Eng..

[35] Gu J., Ren C., Zong X., Chen C., Winnubst L. (2016). Preparation of alumina membranes comprising a thin separation layer and a support with straight open pores for water desalination. Ceram. Int..

[36] Roustaei A., Gosselin A., Frigaard I.-A. (2015). Residual drilling mud during conditioning of uneven boreholes in primary cementing. Part 1: Rheology and geometry effects in non-inertial flows. J. Non-Newton. Fluid.

[37] Yan K., Guan Z., Chen H., Zhao X., Zhang H. (2016). A new calculation method of casing external squeezing loads in complex formation. Eng. Comput..

[38] Gao S., Xu S. (2007). Experimental research on tension property of polyvinyl alcohol fiber reinforced cementitious composites. J. Dalian Univ. Technol..

